# Epidemiology of SLE in Italy: an observational study using a primary care database

**DOI:** 10.1136/lupus-2024-001162

**Published:** 2024-05-13

**Authors:** Pietro Ferrara, Ippazio C Antonazzo, Manuel Zamparini, Carla Fornari, Cristiana Borrelli, Silvia Boarino, Alessandra Bettiol, Irene Mattioli, Pasquale Palladino, Elena Zanzottera Ferrari, Giacomo Emmi, Lorenzo G Mantovani, Giampiero Mazzaglia

**Affiliations:** 1 Center for Public Health Research, University of Milan-Bicocca, Monza, Italy; 2 Laboratory of Public Health, Istituto Auxologico Italiano Istituto di Ricovero e Cura a Carattere Scientifico, Milano, Italy; 3 Medical Affairs Department, AstraZeneca, Milan, Italy; 4 Department of Experimental and Clinical Medicine, University of Florence, Firenze, Italy; 5 Cegedim Health Data, Milan, Italy; 6 Department of Medicine - Centre for Inflammatory Diseases, Monash University, Clayton, Victoria, Australia

**Keywords:** Systemic Lupus Erythematosus, Epidemiology, Prevalence, Incidence

## Abstract

**Objectives:**

To estimate the incidence and prevalence of SLE in Italy, and to describe the demographic and clinical characteristics of patients with newly diagnosed SLE.

**Methods:**

A retrospective cohort study was conducted using The Health Improvement Network general practice database in Italy, encompassing data from 634 753 people. SLE cases were identified over the period 2017–2022, employing three alternative definitions to provide a more detailed understanding of SLE characteristics. Incidence rates were expressed as cases per 100 000 person-years and prevalence as cases per 100 000 people. Demographic and clinical characteristics of incident SLE cases were also studied.

**Results:**

From 2017 to 2022, a total of 191 incident and 1385 prevalent cases were identified under our first definition. In 2022, the incidence rate was 6.51 cases (95% CI 6.29 to 6.74) per 100 000 person-years, and the prevalence 60.57 (95% CI 59.89 to 61.25) per 100 000 people, being the prevalence five times higher in women compared with men. Both estimates have trended upwards since 2017. A geographical variation across the country was also seen. The demographic and clinical characteristics of incident SLE cases were described, while the potential associations of SLE incidence with some pre-existing conditions were observed, such as chronic kidney disease, chronic hepatic disease, rheumatoid arthritis and Sjogren’s syndrome.

**Conclusions:**

The results of this nationwide study, the first conducted in Italy, showed that the incidence of SLE has increased in Italy in recent years. Age, sex, and area of residence strongly correlate with the epidemiology of this condition.

**WHAT IS ALREADY KNOWN ON THIS TOPIC**

- Exploring the epidemiology of SLE poses challenges because of variations in case definitions over time, differences in the source population (such as community vs hospital settings) and disparities in data sources (such as hospital charts and healthcare administrative data).
- Currently, a comprehensive epidemiological overview of this condition is lacking in Italy.

**WHAT THIS STUDY ADDS**

- Using a nationwide primary care database, this study successfully estimated the incidence and prevalence of SLE throughout Italy.
- The analysis with controls extracted from the same database allowed profiling of the demographic and clinical characteristics of newly diagnosed cases of SLE.

**HOW THIS STUDY MIGHT AFFECT RESEARCH, PRACTICE OR POLICY**

- Results from this study can be exploited to design strategies and management guidelines for SLE in Italy to enhance patient care and reduce the clinical and resource burden associated with this condition.

## Introduction

 SLE is an autoimmune disease with a relapsing and remitting course featuring a wide spectrum of clinical manifestation, from mild to life-threatening conditions.^1^,^3^ The heterogeneity and severity of the clinical manifestations may also vary across different ethnic groups, making the diagnosis of SLE particularly challenging.

As a result, the most recent studies that estimated incidence and prevalence of SLE have shown considerable variation across different geographical areas. In a recent systematic review incidence ranged from 0.3 to 8.7 per 100 000 person-years and prevalence from 3.2 to 159 per 100 000 people.^4^ In Europe, the overall incidence of SLE varies from 1.5 to 7.4 per 100 000 person-years,^5^,^7^ whereas the estimated prevalence of SLE between 29 and 210 per 100 000 people.^5 8^ In Italy, studies conducted in limited geographical contexts observed that the incidence and prevalence of SLE are among the lowest identified in Europe, ranging between 2.0 and 2.6 per 100 000 person-years and 39.2 and 81 per 100 000 people, respectively.^9^,^13^

Such variability is likely to be attributed to differences of population structure, ethnicity and the distribution of environmental factors. However, it can also be explained by differences in study design, data source and sample size, and difficulties in developing optimal case definition.

Recently, a study conducted in the UK using the Clinical Practice Research Datalink estimated an SLE incidence of 4.9 per 100 000 person-years and a prevalence of 97 per 100 000 people.^14^ Patient data—encompassing demographics, diagnoses, specialist visits, laboratory results and drug therapies—facilitated the creation of diverse SLE definitions. These ranged from basic diagnostic codes to complex algorithms considering the SLE classification criteria identified by the European League Against Rheumatism and the American College of Rheumatology (EULAR/ACR).^15 16^

Therefore, general practice (GP) databases represent a reliable source of healthcare data that might be explored to assess the epidemiology of SLE in a population-based setting. The objective of this study was to investigate the incidence and prevalence of SLE in Italy from 2017 to 2022. Additionally, the study aimed to profile the newly diagnosed cases of SLE in terms of demographic and clinical variables in comparison to a control group.

## Methods

### Study design and data source

A retrospective cohort study was conducted using data from The Health Improvement Network (THIN) database, a large standardised European database network of fully anonymised longitudinal primary care electronic health records. Within THIN, anonymised data regarding patients’ health and treatments are transmitted by the GPs who have joined the THIN network in Europe. THIN and access to the data are also overseen by an independent advisory committee consisting of clinicians, researchers and patients. For this study, data were retrieved from the Italian THIN database, which collectss longitudinal anonymised patient-level information on health events and healthcare resources reimbursed by the National Health Service. It encompasses approximately 1 million active patients with an average of around 7 years of clinical data history, registered with over 550 Italian GPs distributed over the whole country. Medical events are recorded by GPs and coded using the International Classification of Disease, Ninth Revision, Clinical Modification (ICD9-CM) classification. Prescription data are automatically recorded each time a GP issues a prescription coded using the national formulary. Data regarding contact with secondary care are inputted from referrals and discharge letters. Numerous published reports have showcased the accuracy and comprehensiveness of the information from the GP database within the THIN network regarding patients’ demographics, prevalence and mortality rates of chronic conditions.^17^,^19^

### Study population

The study population comprised the general adult (≥18 years) population registered in the patients’ lists of Italian GPs who agreed to participate in the Italian THIN network. For the purpose of this research, a total of 634 753 eligible individuals were identified, contributing data between 1 January 2017 and 31 December 2022, with at least one contact with a GP for any medical or administrative reason (ie, the entry date) and with an available follow-back of at least 3 years. The patient exit date was defined as the date of last data collection, transfer-out from a participating GP or death, whichever comes first.

We also computed the size of the overall population that we would have needed to observe in order to reach a prespecified level of precision of SLE estimates, using the following formula:^20^

$$n=\frac{Z_{1-\frac{\alpha }{2}}^{2}P\left(1-P\right)}{d^{2}}$$

where *P* represents the assumed prevalence, *d* the level of precision and *α* the type I error. Assuming a SLE prevalence of 81 per 100 000 in Italy based on the literature,^9^,^13^ a population of 310 918 individuals provides a level of precision of 0.01% in the estimate.

### Case definition

To gain a deeper insight into the characteristics of SLE, the study considered three distinct definitions (detailed in online supplemental table S1 and figure S1): our first definition encompassed (1) Systemic lupus or individuals meeting two or more criteria of an adapted version of the EULAR/ACR classification; the other two alternative definitions considered (2) Only systemic lupus, and (3) A comprehensive definition encompassing all forms of lupus, including cutaneous-only lupus.

The first definition of ‘full systemic lupus’ includes the diagnostic codes representing SLE or a subtype of SLE but excludes cutaneous-only lupus. In addition, the first definition also included subjects meeting two or more criteria for SLE as defined by an adapted version of the EULAR/ACR criteria, but only if they had at least one prescription of medication commonly used for SLE or positive results on laboratory tests within 6 months after the index diagnosis.

The rationale for using an adapted version of the EULAR/ACR classification is due to the fact that, considering how the databases of GPs in Italy are constructed, it should be noted that certain criteria items of the EULAR/ACR (eg, some laboratory test results) may not be recorded in primary care databases. The second definition of ‘systemic lupus code only’ includes diagnostic codes representing SLE or a subtype of SLE while excluding cutaneous-only lupus. The third definition of ‘fully comprehensive lupus’ includes subjects selected under the second definition, as well as those with cutaneous-only lupus or those meeting two or more criteria for SLE as defined by the adapted version of the EULAR/ACR criteria, but only if they had at least one prescription of medication commonly used for SLE or positive results to laboratory tests within 6 months after the index diagnosis. The index date (ID) was considered as the first date of registration of codes related to the inclusion criteria.

Exclusion criteria were applied for patients identified using the diagnostic code for SLE or cutaneous-only lupus if the following diseases were retrieved prior to the ID: primary vasculitis, myositis, polymyositis, dermatomyositis, psoriatic arthritis, CREST syndrome (Calcinosis, Raynaud’s phenomenon, Esophageal dysmotility, Sclerodactyly and Telangiectasia) or scleroderma.^21^

### Covariates and matched control analysis

To characterise patients with a new diagnosis of SLE, the presence of the following covariates was evaluated at the ID: (1) Demographic data (age, sex and geographical area); (2) Presence of EULAR/ACR criteria; (3) Comorbidities (ie, diabetes, chronic kidney disease (CKD), cardiovascular diseases, cerebrovascular accident, hypertension, dementia or Alzheimer’s disease, Parkinson’s disease, mood and anxiety disorders, chronic hepatic diseases, osteoporosis, malignancy, chronic obstructive pulmonary disease, autoimmune diseases (including multiple sclerosis, rheumatoid arthritis, inflammatory bowel diseases, ankylosing spondylitis, myasthenia gravis, Sjogren’s syndrome)) identified as detailed in online supplemental table S1; (4) Number of concomitant therapies (Anatomical Therapeutic Chemical classification system V level) in the 6 months before the ID; (5) Clinical parameters, such as body mass index and blood pressure (ie, the last value recorded within 3 years prior to the ID).

To identify potential characteristics associated with SLE diagnosis and analyse comorbidity prevalence in incident SLE cases, patients were compared with four non-SLE controls. Controls for each SLE case were randomly extracted from the same data set, which included all the 634 753 individuals registered in the Italian THIN database and were matched by year of birth and sex.

### Study outcomes and analysis

Prevalent cases were considered as all living cases of SLE who met the inclusion and exclusion criteria for the alternative SLE definitions on 31 December of each study year. Annual prevalence has been expressed as the number of cases per 100 000 people with 95% CI, calculated by dividing the number of prevalent cases by the number of active patients from the source population included in the THIN database on 31 December of each study year.

Incident cases were considered all living cases of SLE on the database who met for the first time the inclusion and exclusion criteria for the alternative SLE definitions within each study year. The incidence has been expressed as the number of cases per 100 000 person-years (with 95% CI) and calculated by dividing the number of incident cases by the number of person-years from eligible patients in the source population during the study period. For each year of follow-up, the entry date was set on 1 January, or the ID. The exit date was the earliest date of incident SLE diagnosis, death, transfer-out from a participating GP, last data collection or 31 December of the specified year. Standardised prevalence/incidence estimates were evaluated by computing the age-specific and sex-specific prevalence/incidence and by using the 2022 ≥18-year-old Italian population as reference.

Incident cases of SLE (ie, full systemic lupus) and matched controls (ie, age-matched and sex-matched individuals from the same database) were selected to describe the demographic and clinical characteristics of patients with SLE and assess potential association with SLE diagnosis. All categorical data were summarised through frequency and percentage, while continuous variables were described using mean and SD. Differences between categorical variables were evaluated through χ^2^ and Fisher’s exact tests. Student’s t-test was used to assess differences between continuous variables.

Univariable and multivariable logistic regression models were used to investigate the association of SLE diagnosis with patients’ demographic and clinical characteristics. In the multivariable model, age and sex were included as fixed variables, while other variables were included only if they showed significant results in the univariable analysis (p≤0.05). Results were expressed as ORs with 95%CIs. Statistical analyses were conducted using R V.4.0.5 (R Foundation for Statistical Computing, Vienna, Austria) and SAS V.9.4 (SAS Institute, Cary, North Carolina, USA).

## Results

Over the entire study period, a total of 191 incident cases and 1385 prevalent cases were identified in the study population using the first definition. Table 1 reports data on incident and prevalent cases of SLE over the years 2017–2022, while the results for the alternative definitions are shown in online supplemental tables S2 and S3. SLE cases showed a gradual increase in the standardised incidence rates from 4.99 (95% CI 4.79 to 5.18) per 100 000 person-years in 2017 to 6.51 (95% CI 6.29 to 6.74) in 2022. It peaked significantly in 2021, reaching a level two times higher than that of the previous year, and then returned to the previous trend. Prevalence reported a steadier linear increase, rising from 36.04 (95% CI 35.51 to 36.57) per 100 000 individuals in 2017 to 60.57 (95% CI 59.89 to 61.25) in 2022. Both estimates were higher than those under the second definition, which reported an incidence of 6.16 (95% CI 5.94 to 6.38) and a prevalence of 54.94 (95% CI 54.29 to 55.59) in the year 2022, but lower than the third definition, which estimated incidence and prevalence in 2022 at 9.67 (95% CI 9.40 to 9.95) and 74.20 (95% CI 73.44 to 74.66). All the alternative definitions reported similar trends in terms of year, age, sex and geographical location (online supplemental tables S2 and S3).

**Table 1:** SLE incidence rate (per 100 000 person-years) and prevalence (per 100 000 people) by year, 2017–2022 (first definition)

Year	Incidence rate				Prevalence		
	Incident cases	Person-years	Crude(95% CI)	Standardised* (95% CI)	Prevalent cases	Crude(95% CI)	Standardised*(95% CI)
2017	25	472 862	5.29(3.21 to 7.36)	4.99(4.79 to 5.18)	176	37.22(31.72 to 42.72)	36.04(35.51 to 36.57)
2018	28	482 920	5.80(3.65 to 7.95)	5.46(5.25 to 5.66)	198	41.00(35.29 to 46.71)	39.75(39.2 to 40.3)
2019	23	491 726	4.68(2.77 to 6.59)	4.46(4.27 to 4.65)	216	43.93(38.07 to 49.78)	42.73(42.15 to 43.3)
2020	26	495 645	5.25(3.23 to 7.26)	5.07(4.87 to 5.27)	233	47.01(40.97 to 53.04)	46.13(45.53 to 46.73)
2021	58	497 692	11.65(8.65 to 14.65)	11.19(10.89 to 11.48)	281	56.46(49.86 to 63.06)	55.60(54.95 to 56.26)
2022	31	458 820	6.76(4.38 to 9.13)	6.51(6.29 to 6.74)	281	61.24(54.09 to 68.4)	60.57(59.89 to 61.25)

* By age and sex, year 2022 Italian population as reference.

**Figure 1:**
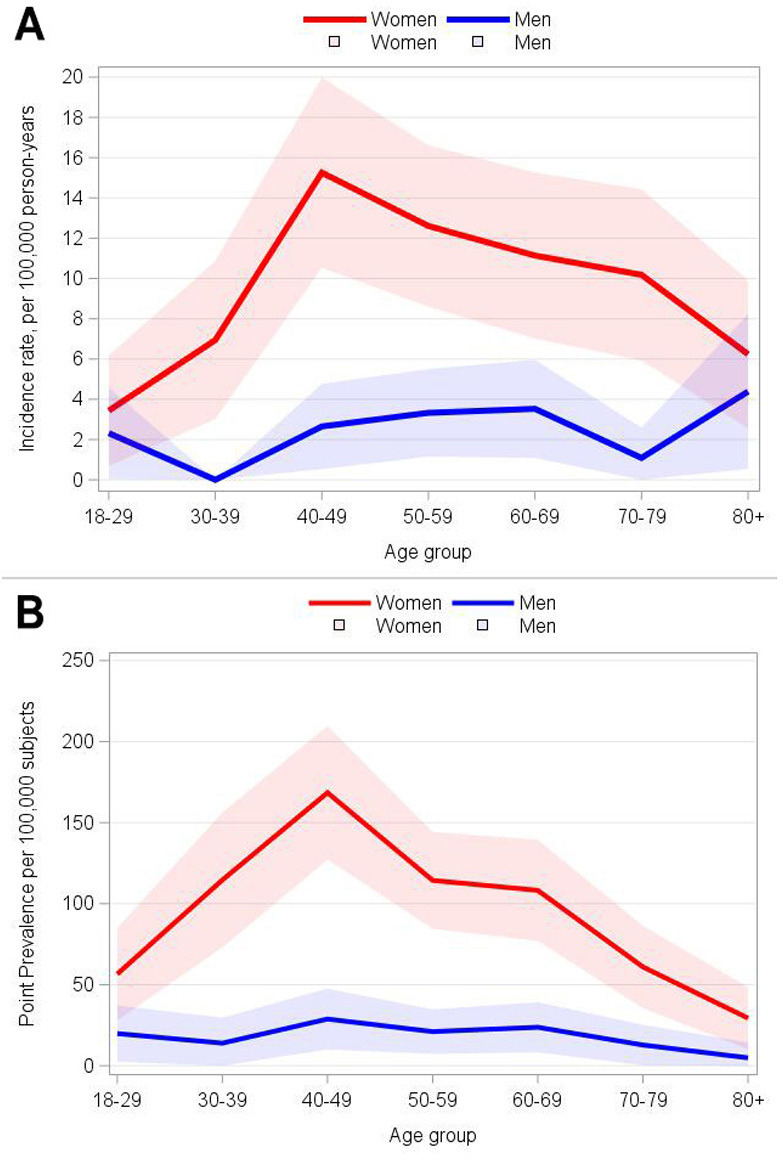
Line chart of sex-specific and age-specific incidence rate (2017–2022; A) and point prevalence (2022; B) for SLE. The lines represent the estimates, and the bands indicate the 95% CIs.

In 2022, the incidence was highest among women in the 40–49 age group, while in men, the peak occurred at a later age. Additionally, it was greater in women compared with men for all ages (figure 1A). Throughout all study years, prevalence was higher in women than men, with a ratio of approximately 5 to 1 (figure 1B).

Table 2 describes clinical and demographic characteristics of incident cases of SLE compared with age-matched and sex-matched controls. Mean age at diagnosis was 55.9 years and women accounted for 82.10% of SLE cases. Incidence exhibited geographical variation, being highest in Northern Italy (44.74%) and lowest in Southern Italy and the Islands (20.53%). Overall, at the time of diagnosis, 6.84% of cases had already registered at least one SLE symptom according to EULAR/ACR criteria, compared with only 0.13% (one patient) in the control group. Compared with controls, subjects with SLE had a higher prevalence of certain comorbidities, such as CKD (5.79% vs 1.45%), chronic hepatic disease (6.32% vs 1.97%) and osteoporosis (16.32% vs 9.47%), as well as concomitant autoimmune diseases, including rheumatoid arthritis (5.79% vs 1.97%) and Sjogren’s syndrome (3.68% vs 0.39%).

**Table 2:** Demographic and clinical characteristics of incident SLE cases

	SLE cases	Controls	P value
	N (%)	N (%)	
Overall	**190**	**760**	
Sex			
Male	34 (17.90)	136 (17.90)	–
Female	156 (82.10)	624 (82.10)	
Age, years, mean (SD)	55.94 (±15.66)	55.94 (±15.63)	–
Age class, years			
18–29	10 (5.26)	40 (5.26)	–
30–39	12 (6.32)	48 (6.32)	–
40–49	46 (24.21)	184 (24.21)	–
50–59	46 (24.21)	184 (24.21)	–
60–69	36 (18.95)	144 (18.95)	–
70–79	24 (12.63)	96 (12.63)	–
80+	16 (8.42)	64 (8.42)	–
Geographical area			
Northern Italy	85 (44.74)	294 (38.68)	0.0318
Central Italy	66 (34.74)	233 (30.66)	
Southern Italy and the Islands	39 (20.53)	227 (29.87)	
SLE symptoms (EULAR/ACR criteria)	13 (6.84)	1 (0.13)	**<**0.0001
Comorbidities			
Diabetes	11 (5.79)	75 (9.87)	0.0797
Chronic kidney disease	11 (5.79)	11 (1.45)	0.0004
Cardiovascular disease	12 (6.32)	56 (7.37)	0.6147
Cerebrovascular accident	10 (5.26)	26 (3.42)	0.2343
Hypertension	82 (43.16)	286 (37.63)	0.1619
Dementia/Alzheimer’s disease	4 (2.11)	7 (0.92)	0.1723
Parkinson disease	2 (1.05)	6 (0.79)	0.7226
Mood and anxiety disorders	35 (18.42)	117 (15.39)	0.3088
Chronic hepatic disease	12 (6.32)	15 (1.97)	0.0013
Osteoporosis	31 (16.32)	72 (9.47)	0.0067
Malignancy	33 (17.37)	106 (13.95)	0.2327
Chronic obstructive pulmonary disease	15 (7.89)	38 (5.00)	0.1200
Concomitant autoimmune disease*	27 (14.21)	38 (5.00)	**<**0.0001
Multiple sclerosis	2 (1.05)	2 (0.26)	0.1328
Rheumatoid arthritis	11 (5.79)	15 (1.97)	0.0039
Inflammatory bowel disease	4 (2.11)	14 (1.84)	0.8119
Ankylosing spondylitis	3 (1.58)	4 (0.53)	0.1292
Myasthenia gravis	2 (1.05)	1 (0.13)	0.1037
Sjogren’s syndrome	7 (3.68)	3 (0.39)	**<**0.0001
Body mass index (mean (SD))†	27.26 (±5.01)	27.12 (±6.41)	0.9057
>30	10 (5.26)	41 (5.39)	0.9974
Concomitant therapies			
of different ATC V levels (mean±SD)	6.31 (±5.25)	4.84 (±4.10)	**<**0.0001

* At least one.

† On total subjects with at least one registered value.

ATC V, Anatomical Therapeutic Chemical classification system VEULAR/ACR, European League Against Rheumatism/American College of Rheumatology

The results of the adjusted multivariate analyses confirmed that, at the time of SLE diagnosis, patients had higher significant odds of being previously diagnosed with CKD (OR 3.88; 95% CI 1.62 to 9.26), chronic hepatic disease (OR 2.93; 95% CI 1.31 to 6.59), rheumatoid arthritis (OR 2.55; 95% CI 1.09 to 5.95), Sjogren’s syndrome (OR 6.66; 95% CI 1.63 to 27.29), as well as a higher odds of being prescribed with five or more concomitant therapies (OR 1.47; 95% CI 1.05 to 2.05) (table 3).

**Table 3:** Crude and multivariable logistic regression analyses of the association between SLE and selected characteristics*

	Crude OR(95% CI)	Adjusted OR*(95% CI)
Geographical area		
Northern Italy	1.68 (1.11 to 2.55)	1.73 (1.21 to 2.67)
Central Italy	1.65 (1.07 to 2.55)	1.62 (1.03 to 2.54)
Southern Italy and Islands	1 (Ref)	1 (Ref)
Comorbidities†		
Chronic kidney disease	4.18 (1.79 to 9.81)	3.88 (1.62 to 9.26)
Chronic hepatic disease	3.35 (1.54 to 7.27)	2.93 (1.31 to 6.59)
Osteoporosis	1.86 (1.18 to 2.94)	1.44 (0.89 to 2.33)
Concomitant autoimmune diseases		
Rheumatoid arthritis	3.05 (1.38 to 6.76)	2.55 (1.09 to 5.95)
Sjogren’s syndrome	9.65 (2.47 to 37.68)	6.66 (1.63 to 27.29)
Concomitant therapies‡	1.65 (1.19 to 2.27)	1.47 (1.05 to 2.05)

SLE due to data absence.

* Adjusted by geographical area, chronic kidney disease, chronic hepatic disease, osteoporosis, rheumatoid arthritis, Sjogren’s syndrome, concomitant therapies.

† Reference category: absence of disease.

‡ Five or more distinct medications; reference category: 0–4 distinct medications.

Ref, reference category

## Discussion

Our nationwide population-based study uses consistent definitions for SLE based on internationally recognised criteria. By extracting data from contemporary primary care databases, we were able to provide robust estimates of the incidence and prevalence of SLE in the adult Italian population. Overall, from 2017 to 2022, SLE exhibited increasing trends in both men and women. However, the burden of this condition is significantly greater in women, with a prevalence approximately five times that of men. The trends were consistent across the three alternative definitions considered. In this sense, our research aligns with several prior reports indicating a global rise in the incidence and prevalence of SLE.^22 23^

Under the first definition, we estimated a standardised incidence rate of SLE of 6.51 (95%CI 6.29 to 6.74) per 100 000 person-years and a standardised point prevalence 60.57 (95%CI 59.89 to 61.25) per 100 000 people in 2022. Literature on incidence and prevalence trends of SLE in Italy is limited. Comparisons are challenging due to a lack of uniformity in case definitions, differences in the source population (eg, community vs hospital setting) and disparities in data sources (eg, hospital charts, healthcare administrative data). Moreover, many studies are confined to regional or even provincial contexts.^9^,^12^

It is essential to highlight the observed increase in SLE incidence in 2021, more than doubling compared with the previous year. Although it decreased again in 2022, it remained consistently higher compared with the initial years in the study. Various hypotheses can explain this peak in incidence. It may be linked to the implementation of the new EULAR/ACR classification criteria developed at the end of 2019. The suggested differential impact of these classification criteria on SLE estimates is thought to be associated with the ability of these criteria to classify patients at an earlier stage. This is in addition to disparities in sensitivity and comparative specificity of EULAR/ACR criteria when compared with other classification systems.^24 25^ A possible impact of COVID-19 should also be considered. While the pandemic did not affect the estimates of the burden of diseases for 2020, population-wide research conducted in the USA and Hong Kong has linked COVID-19 with an increased risk of autoimmune diseases.^26 27^ Regarding SLE, a risk approximately three times higher was in fact observed in the COVID-19 cohort.^27^

In line with previous research,^1428^,^31^ our analysis confirms that SLE incidence and prevalence are higher in women than in men, with the latter experiencing a later peak. The well-established greater susceptibility of women to autoimmunity, and consequently to SLE, is correlated with sex hormones and X linked genetic factors. The female-to-male ratio in SLE can reach values of 9:1, depending also on the correlation between age and the actions of other factors involved in the pathogenesis of SLE.^32 33^ For instance, an even higher female predominance is observable during peak reproductive years, with incidence rates that can reach 8:1–15:1 when compared with age-matched men; these rates fall to 3:1–5:1 in the preadolescent population and after menopause, when oestrogen levels are more similar between sexes.^34^ Variabilities among epidemiological reports, differences in the age distribution of our cohort, as well as possible differences compared with cohorts built on hospital data versus primary care health records, may explain the small differences in the observed female-to-male ratio.

According to several epidemiological studies, indeed, age is an independent factor in the onset and diagnosis of SLE. While SLE can develop in people of all ages, the risk is particularly high in women of childbearing age (15–44 years), while an older age at diagnosis is more typical in men.^35 36^ Our analyses depict the increase in incidence in women as a function of age, peaking in the 40–49 years age group. In our study, the average age at SLE diagnosis and the peak age of incidence are later than those typically taught and observed in previous Italian reports;^37^ however, they align with findings from previous international studies, including analyses that estimated the incidence and prevalence of SLE through GP records.^14 38 39^ The reasons for this higher incidence in middle-aged patients are likely multifactorial and warrant further investigation. One possible explanation could be a pool of previously undetected/misdiagnosed patients that is not captured in the databases of clinical centres and hospitals,^30^ resulting in delayed diagnosis in primary care settings, also due to increasing awareness among GPs as suggested by Jonsson *et al*.^39^ Furthermore, changes in nutritional and environmental exposures may underlie a real increase in older age groups.^39 40^

There was regional variation in incidence and prevalence of SLE in Italy. While a precise pattern over time cannot be described among the three different areas considered, we observed a higher burden in the northern regions in the last 2 years under analysis, consistently across the three alternative definitions considered. The higher prevalence in certain areas cannot be attributed to a single cause, as SLE is a multifactorial condition, with pathogenesis involving genetic and environmental factors, and its burden involving factors related to ethnicity, as well as healthcare access and quality.^2 31 41^ Interestingly, a comprehensive systematic analysis and modelling study on the global epidemiology of SLE has clearly demonstrated an unequal distribution among geographical regions.^42^ These differences have also been found within national territories in several countries, including the UK, the USA, Canada and China.^14 38 43 44^ Possible explanations may include differences in variations in age, genetic and ethnic distribution within the population, income of population groups, disparities in health-seeking behaviour, local environmental exposures (including climatic-meteorological conditions), and the management of clinical databases across different regions.^1441 42 44^,^46^ Further research on geographical variation of SLE is needed.

Significant associations were found between SLE and CKD, chronic hepatic disease and osteoporosis, as well as other concomitant autoimmune diseases when comparing patients with SLE to controls. Many comorbidities are commonly identified at the time of diagnosing SLE, and the burden of comorbidity is high in patients with SLE.^47^ Kidney injury is a typical finding in patients with SLE, due to immune complexes accumulating in glomeruli, resulting in lupus nephritis and facilitating the development of CKD. The latter is a significant risk factor for worsened morbidity and mortality in SLE.^48 49^ Liver involvement is also common in SLE, including both autoimmune mechanisms and non-autoimmune liver diseases, as well as drug-induced toxicity. Often, liver disease in SLE has multifaceted manifestations and origins, and the study of the association and outcomes between SLE and hepatic disease deserves greater attention.^50^ Studies of patients with SLE, especially women, found an increase in bone loss and osteoporosis compared with healthy controls. Furthermore, the risk of osteoporotic fractures has been related to the presence of nephritis and the use of glucocorticoids.^51 52^ Autoimmune diseases frequently co-occur as they share common pathological pathways or genetic aetiology. In patients with SLE, the presence of rheumatoid arthritis and Sjogren’s syndrome is indeed frequent, consistent with our analyses that have highlighted a higher association compared with controls.^53^ Other possible concomitant autoimmune diseases include autoimmune thyroiditis, Crohn’s disease, and others, although in our cohort there was no noticeable higher prevalence compared with controls. This may be due to differences in the traceability of comorbidities through the ICD9-CM code, which may not be accurately reported by all GPs contributing to the Italian THIN database. It should also be considered that the coexistence of SLE and other autoimmune conditions is a diagnostic challenge because there can be an overlap of many clinical manifestations and signs that could mask the concurrent diagnosis in patients recently diagnosed with SLE. Overall, early diagnosis and management of comorbidities and concomitant diseases are crucial to improving the outcomes of patients with SLE.^47^

Potential limitations to the present study warrant discussion. First, the exclusive reliance on GP data, rather than using linked primary and secondary care data, may result in an underestimation of the incidence and prevalence of SLE, especially because certain criteria items of the EULAR/ACR classification (eg, some laboratory test results) may not be recorded in the Italian THIN database. This is why an adapted version of the EULAR/ACR criteria had to be used. However, most of the diagnoses and conditions considered for case definitions are well recorded in the Italian primary care database. Moreover, the use of three alternative definitions allows for estimating the epidemiology of SLE under different scenarios. Second, although over 630 000 data points, sourced from over 550 GPs across the entire country, are sufficient to provide accurate epidemiological estimates of SLE in Italy, it should be noted that future analyses based on a larger population sample may help refine the estimates presented in this study. Third, the incidence estimates may be affected by the small number of cases identified in different years, which could explain the fluctuations over time and the ratios between the various analysed categories, as also reflected in the wide estimated CIs. The association with prevalence estimates, which showed lower degree of uncertainty, however, mitigated this limitation and helped provide a more comprehensive picture of the burden of SLE in Italy. Fourth, GP databases in Italy lack of paediatric information since subjects aged less than 18 years are cared by family paediatricians. Considering that paediatric-onset SLE represents 10%–20% of all SLE cases,^54^ the total prevalence of SLE would increase to around 67 cases per 100 000, corresponding to approximately 33 000 cases in the whole Italian population. Fifth, it is worth emphasising that our study primarily involves descriptive analyses of SLE. Regarding the study of concurrent comorbidities or autoimmune diseases, the analyses only included diagnoses recorded before the ID identified for SLE: thus, the prevalence of conditions associated with lupus may have been underestimated for some patients. Lastly, the order of diagnoses is based on the date of registration, which might not precisely correspond to the date of the initial diagnosis, preventing the establishment of causal and temporal directionalities in the relationship between SLE and concurrent diseases.

In conclusion, our findings reveal a significant burden of SLE in Italy, with incidence and prevalence of SLE depicting rising trends. Analyses by sex and age demonstrate that the burden is significantly higher in women than in men, with incidence beginning to increase in young adults and reaching its peak in middle-age groups. We observed a gradient among different macro-areas of the country, which warrants further research. Considering that SLE impacts the quality of life of patients and is associated with significant healthcare resource consumption, this study provides valuable insights for developing strategies to improve health outcomes and reduce costs associated with the increasing burden of SLE.

## supplementary material

10.1136/lupus-2024-001162 online supplemental file 1

## Data Availability

Data are available upon reasonable request.
