# Supplementary material for: Epidemiology of SLE in Italy: an observational study using a primary care database
Source: Lupus Sci Med. 2024 May 13;11(1):e001162. doi: 10.1136/lupus-2024-001162 (PMC11097856; doi:10.1136/lupus-2024-001162)

SUPPLEMENTARY MATERIALS

Table S1. List of diagnosis for disease identification and patients' characterisation

	**ICD9-CM**
Main inclusion criteria	
Systemic lupus erythematosus	710.0
Cutaneous only lupus	695.4, 373.34
EULAR/ACR criteria	
Leukopenia	288.50, 288.59
Thrombocytopenia	287.3, 287.4, 287.5
Autoimmune haemolysis	283.0
Delirium	297
Psychosis	290-299
Seizure	333.2, 345.0-345.9
Non-scarring alopecia	704.0
Oral ulcers	528.0
Acute pericarditis	420
Pleural and pericardial effusion	510.9, 511, 391.0, 393,423.1, 423.2
Exclusion criteria	
Primary vasculitis	446, 447.5
Myositis	729.1
Polymyositis	710.4
Dermatomyositis	710.3
Psoriatic arthritis	696.0
CREST syndrome or scleroderma	710.1
Comorbidity	
Diabetes	250
Chronic kidney disease	582, 585, 586
Cardiovascular disease (i.e., myocardial infarction, coronary artery bypass graft, unstable angina, angina pectoris, peripheral artery disease, heart failure, atrial fibrillation)	402.01, 402.11, 402.91, 410, 411, 413, 414.0 (exc. 414.02–07), 414.02–07, V45.81–82, 440.2, 433.9, 444.2, 427.3, 428
Cerebrovascular accident (i.e., stroke and transient ischemic attack)	430–436, V12.54
Hypertension	401–405, 997.91
Dyslipidaemia	272
Dementia/Alzheimer's disease	290, 294.1, 331.2
Parkinson's disease	332
Mood and anxiety disorders	300.0, 300.2, 296.0, 296.1, 296.04, 296.14, 296.4, 296.44, 296.5, 296.54, 296.6, 296.7, 296.8, 296.2, 296.3, 298.0, 300.4, 311
Chronic hepatic disease	456.0–456.2; 571–573
Autoimmune disease	
Multiple sclerosis	340
Rheumatoid arthritis	714.0, 714.1, 714.2, 714.30, 714.32, 714.33,
Inflammatory bowel disease	564.1
Ankylosing spondylitis	720

Idiopathic thrombocytopenic purpura	287.31
Myasthenia gravis	358.0
Sjogren's syndrome	710.2
Osteoporosis	733
Malignancy	140–239
Chronic obstructive pulmonary disease	490, 491, 492, 494, 496

Abbreviations: ICD9-CM, International Classification of Disease, Ninth Revision, Clinical Modification classification; EULAR/ACR, European League Against Rheumatism and American College of Rheumatology.

Table S2. Annual (2017–2022) standardised incidence rate (per 100,000 person-years) of SLE, stratified by sex, age, and geographical area.

Variable		2017	2018	2019	2020	2021	2022
**First definition**							
Overall		4.99 (4.79–5.18)	5.46 (5.25–5.66)	4.46 (4.27–4.65)	5.07 (4.87–5.27)	11.19 (10.89–11.48)	6.51 (6.29–6.74)
Sex	*Female*	9.08 (5.37–12.80)	8.91 (5.27–12.55)	7.61 (4.27–10.94)	7.54 (4.23–10.84)	18.33 (13.20–23.47)	8.87 (5.16–12.57)
	Male	0.91 (0–2.17)	2.23 (0.27–4.18)	1.31 (0–2.79)	2.61 (0.52–4.69)	3.91 (1.35–6.46)	4.27 (1.48–7.06)
Age	*18–29*	1.73 (0–5.11)	0	1.67 (0–4.93)	3.35 (0–7.99)	6.81 (0.15–13.49)	3.86 (0–9.22)
	30–39	1.77 (0–5.23)	3.56 (0–8.48)	5.39 (0–11.49)	1.83 (0–5.43)	7.52 (0.15–14.88)	2.13 (0–6.32)
	40–49	9.22 (2.83–15.61)	9.27 (2.85–15.69)	5.89 (0.73–11.06)	12.16 (4.63–19.70)	11.35 (3.94–18.77)	8.66 (1.73–15.60)
	50–59	8.78 (2.70–14.86)	5.29 (0.65–9.93)	3.09 (0–6.59)	7.12 (1.85–12.40)	18.15 (9.77–26.54)	6.54 (1.31–11.77)
	60–69	2.65 (0–6.32)	7.77 (1.55–13.99)	6.28 (0.78–11.78)	3.68 (0–7.85)	11.89 (4.52–19.27)	12.43 (4.73–20.13)
	70–79	3.21 (0–7.67)	4.63 (0–9.87)	7.48 (0.92–14.04)	1.46 (0–4.33)	14.22 (5.41–23.03)	4.45 (0–9.48)
	80+	7.02 (0–14.97)	8.94 (0.18–17.7)	2.09 (0–6.20)	3.92 (0–9.36)	5.67 (0–12.08)	5.85 (0–12.47)
Geographical area	*Northern Italy*	3.28 (3.05–3.52)	5.00 (4.72–5.29)	4.62 (4.35–4.90)	6.33 (6.01–6.66)	12.40 (11.95–12.86)	7.55 (7.19–7.90)
	Central Italy	7.92 (7.36–8.47)	7.69 (7.14–8.23)	4.86 (4.43–5.30)	3.63 (3.26–4.01)	10.76 (10.12–11.41)	5.03 (4.59–5.47)
	Southern Italy & Islands	4.04 (3.74–4.35)	3.28 (3.00–3.55)	3.89 (3.59–4.19)	4.8 (4.47–5.13)	9.34 (8.88–9.80)	6.79 (6.40–7.19)
**Second definition**							
Overall		4.58 (4.40–4.77)	5.08 (4.88–5.27)	4.05 (3.87–4.22)	4.82 (4.63–5.02)	10.48 (10.19–10.76)	6.16 (5.94–6.38)
Sex	*Female*	8.69 (5.06–12.32)	8.52 (4.96–12.08)	6.85 (3.68–10.01)	7.54 (4.23–10.84)	16.84 (11.92–21.76)	8.06 (4.53–11.59)

Variable		2017	2018	2019	2020	2021	2022
	Male	0.46 (0–1.35)	1.78 (0.04–3.52)	1.31 (0–2.79)	2.17 (0.27–4.07)	3.91 (1.35–6.46)	4.27 (1.48–7.06)
Age	*18–29*	1.73 (0–5.11)	0	1.67 (0–4.93)	1.68 (0–4.96)	6.81 (0.14–13.49)	3.86 (0–9.22)
	30–39	1.77 (0–5.23)	3.56 (0–8.48)	3.59 (0–8.58)	1.83 (0–5.43)	7.52 (0.15–14.88)	2.13 (0–6.32)
	40–49	9.22 (2.83–15.61)	9.27 (2.85–15.69)	4.72 (0.09–9.34)	12.16 (4.63–19.7)	11.35 (3.94–18.77)	8.66 (1.73–15.6)
	50–59	8.78 (2.7–14.86)	5.29 (0.65–9.93)	3.09 (0–6.59)	7.12 (1.85–12.4)	16.13 (8.23–24.04)	6.54 (1.31–11.77)
	60–69	2.65 (0–6.32)	7.77 (1.55–13.99)	6.28 (0.78–11.78)	3.68 (0–7.85)	11.89 (4.52–19.27)	12.43 (4.73–20.13)
	70–79	1.61 (0–4.75)	3.09 (0–7.37)	7.48 (0.92–14.04)	1.46 (0–4.33)	14.22 (5.41–23.03)	4.45 (0–9.48)
	80+	4.68 (0–11.17)	6.71 (0–14.29)	2.09 (0–6.2)	3.92 (0–9.36)	1.89 (0–5.59)	1.95 (0–5.77)
Geographical area	*Northern Italy*	2.81 (2.6–3.03)	5.00 (4.72–5.29)	3.65 (3.4–3.89)	5.76 (5.45–6.07)	11.97 (11.52–12.41)	7.55 (7.19–7.9)
	Central Italy	7.33 (6.8–7.87)	7.12 (6.6–7.65)	4.86 (4.43–5.3)	3.63 (3.26–4.01)	9.1 (8.5–9.69)	4.47 (4.05–4.88)
	Southern Italy & Islands	4.04 (3.74–4.35)	2.42 (2.18–2.66)	3.89 (3.59–4.19)	4.8 (4.47–5.13)	9.34 (8.88–9.8)	6.07 (5.7–6.45)
**Third definition**							
Overall		6.23 (6.02–6.45)	6.22 (6.01–6.44)	5.02 (4.82–5.22)	5.90 (5.69–6.12)	14.68 (14.34–15.02)	9.67 (9.40–9.95)
Sex	*Female*	10.66 (6.64–14.69)	10.46 (6.51–14.4)	8.75 (5.17–12.32)	9.05 (5.43–12.66)	22.82 (17.1–28.55)	13.3 (8.76–17.84)
	Male	1.82 (0.04–3.61)	2.23 (0.27–4.18)	1.31 (0–2.79)	2.61 (0.52–4.69)	6.08 (2.89–9.26)	6.17 (2.82–9.52)
Age	*18–29*	3.45 (0–8.24)	0	1.67 (0–4.93)	3.35 (0–7.99)	8.52 (1.05–15.98)	3.86 (0–9.22)
	30–39	1.77 (0–5.23)	3.56 (0–8.48)	5.39 (0–11.49)	5.5 (0–11.72)	9.4 (1.16–17.63)	6.4 (0–13.64)
	40–49	10.37 (3.6–17.15)	11.59 (4.41–18.77)	7.07 (1.41–12.73)	12.16 (4.63–19.7)	22.7 (12.21–33.19)	11.55 (3.55–19.56)

**Variable**		**2017**	**2018**	**2019**	**2020**	**2021**	**2022**
	50–59	10.98 (4.17–17.78)	7.41 (1.92–12.9)	3.09 (0–6.59)	9.16 (3.17–15.14)	20.17 (11.33–29.01)	10.90 (4.14–17.65)
	60–69	3.97 (0–8.47)	7.77 (1.55–13.99)	7.53 (1.51–13.56)	3.68 (0–7.85)	14.27 (6.2–22.35)	17.40 (8.29–26.52)
	70–79	4.82 (0–10.27)	4.63 (0–9.87)	8.98 (1.8–16.17)	1.46 (0–4.33)	17.06 (7.41–26.72)	7.42 (0.92–13.92)
	80+	7.02 (0–14.97)	8.94 (0.18–17.7)	2.09 (0–6.2)	3.92 (0–9.36)	5.67 (0–12.08)	7.80 (0.16–15.44)
Geographical area	*Northern Italy*	4.83 (4.54–5.11)	6.34 (6.02–6.67)	5.49 (5.19–5.80)	6.33 (6.01–6.66)	16.6 (16.07–17.12)	11.24 (10.81–11.68)
	Central Italy	9.11 (8.51–9.70)	8.29 (7.72–8.86)	5.42 (4.96–5.87)	4.92 (4.49–5.36)	14.65 (13.89–15.4)	6.98 (6.46–7.50)
	Southern Italy & Islands	4.95 (4.61–5.29)	3.28 (3.00–3.55)	3.89 (3.59–4.19)	6.44 (6.05–6.82)	11.12 (10.61–11.62)	10.41 (9.92–10.90)

Table S3. Annual (2017–2022) standardised prevalence (per 100,000 people) of SLE stratified by sex, age, and geographical area.

**Variable**		**2017**	**2018**	**2019**	**2020**	**2021**	**2022**
**First definition**							
Overall		36.04 (35.51–36.57)	39.75 (39.2–40.3)	42.73 (42.15–43.3)	46.13 (45.53–46.73)	55.6 (54.95–56.26)	60.57 (59.89–61.25)
Sex	*Female*	60.03 (50.49–69.58)	65.84 (55.94–75.73)	70.74 (60.58–80.91)	75.38 (64.94–85.82)	90.54 (79.14–101.9)	97.14 (84.88–109.4)
	Male	10.93 (6.55–15.3)	12.46 (7.85–17.08)	13.11 (8.42–17.8)	14.33 (9.44–19.22)	16.93 (11.61–22.24)	18.98 (13.1–24.86)
Age	*18–29*	22.45 (10.25–34.65)	21.97 (10.03–33.91)	23.32 (11.11–35.54)	26.8 (13.67–39.94)	34.07 (19.14–49)	38.65 (21.71–55.59)
	30–39	47.70 (29.71–65.69)	51.56 (32.8–70.32)	55.72 (36.11–75.33)	58.67 (38.34–78.99)	67.65 (45.56–89.74)	68.28 (44.63–91.93)
	40–49	48.40 (33.77–63.04)	56.77 (40.88–72.66)	63.66 (46.68–80.63)	77.85 (58.78–96.92)	90.8 (69.84–111.7)	105.41 (81.24–129.6)
	50–59	48.29 (34.03–62.56)	51.85 (37.34–66.37)	51.54 (37.26–65.83)	54.94 (40.29–69.59)	69.58 (53.17–85.99)	70.82 (53.61–88.03)
	60–69	34.44 (21.2–47.67)	41.46 (27.1–55.83)	45.2 (30.44–59.96)	46.62 (31.8–61.44)	57.09 (40.95–73.24)	68.36 (50.3–86.42)
	70–79	25.70 (13.11–38.29)	24.7 (12.6–36.81)	29.94 (16.82–43.06)	29.24 (16.43–42.05)	38.39 (23.91–52.87)	38.56 (23.74–53.38)
	80+	18.73 (5.75–31.71)	22.35 (8.5–36.21)	23.03 (9.42–36.63)	17.65 (6.12–29.18)	17.01 (5.9–28.12)	19.5 (7.41–31.58)
Geographical area	*Northern Italy*	42.93 (42.09–43.78)	45.05 (44.19–45.92)	47.93 (47.03–48.82)	52.74 (51.8–53.67)	63.38 (62.35–64.4)	69.93 (68.85–71)
	Central Italy	36.28 (35.1–37.47)	42.95 (41.66–44.24)	46.49 (45.15–47.83)	48.93 (47.55–50.31)	58.99 (57.47–60.5)	62.75 (61.19–64.31)
	Southern Italy & Islands	23.72 (22.98–24.46)	26.38 (25.6–27.16)	29.00 (28.19–29.82)	30.92 (30.08–31.76)	37.26 (36.33–38.18)	41.87 (40.89–42.85)
**Second definition**							
Overall		31.09 (30.6–31.58)	34.96 (34.44–35.48)	37.85 (37.31–38.39)	41.78 (41.21–42.35)	50.55 (49.93–51.18)	54.94 (54.29–55.59)
Sex	*Female*	54.50 (45.41–63.6)	60.42 (50.94–69.89)	64.66 (54.94–74.37)	70.1 (60.03–80.17)	83.81 (72.84–94.78)	89.48 (77.72–101.2)
	Male	6.37	8.01	9.18	10.85	13.45	15.19

Variable		2017	2018	2019	2020	2021	2022
		(3.03–9.71)	(4.31–11.71)	(5.25–13.10)	(6.6–15.11)	(8.72–18.19)	(9.92–20.45)
Age	*18–29*	22.45<br>(10.25–34.65)	21.97<br>(10.03–33.91)	23.32<br>(11.11–35.54)	25.13<br>(12.41–37.84)	32.37<br>(17.81–46.92)	36.72<br>(20.21–53.22)
	30–39	42.4<br>(25.44–59.36)	46.22<br>(28.46–63.99)	48.53<br>(30.23–66.83)	51.33<br>(32.32–70.34)	60.13<br>(39.3–80.96)	59.74<br>(37.62–81.86)
	40–49	47.25<br>(32.79–61.71)	55.61<br>(39.88–71.34)	61.3<br>(44.64–77.96)	75.42<br>(56.65–94.18)	88.28<br>(67.61–108.9)	102.52<br>(78.69–126.3)
	50–59	45.00<br>(31.23–58.77)	48.68<br>(34.62–62.74)	48.45<br>(34.6–62.3)	52.91<br>(38.53–67.28)	64.54<br>(48.73–80.35)	65.37<br>(48.84–81.91)
	60–69	29.14<br>(16.96–41.31)	36.28<br>(22.84–49.72)	40.18<br>(26.26–54.09)	41.71<br>(27.7–55.73)	52.34<br>(36.88–67.8)	63.39<br>(46–80.78)
	70–79	12.85<br>(3.95–21.76)	10.81<br>(2.8–18.81)	17.96<br>(7.8–28.13)	17.54<br>(7.62–27.47)	28.44<br>(15.98–40.9)	29.66<br>(16.66–42.66)
	80+	7.02<br>(0–14.97)	13.41<br>(2.68–24.14)	14.65<br>(3.8–25.51)	15.69<br>(4.82–26.56)	11.34<br>(2.27–20.41)	9.75<br>(1.2–18.29)
Geographical area	*Northern Italy*	37.22<br>(36.43–38.00)	40.41<br>(39.59–41.23)	42.45<br>(41.61–43.29)	47.51<br>(46.62–48.4)	57.63<br>(56.65–58.61)	63.95<br>(62.92–64.98)
	Central Italy	33.74<br>(32.6–34.88)	39.85<br>(38.61–41.09)	43.39<br>(42.1–44.69)	45.83<br>(44.5–47.17)	54.21<br>(52.77–55.66)	57.09<br>(55.6–58.58)
	Southern Italy & Islands	16.75<br>(16.13–17.38)	18.91<br>(18.25–19.56)	22.65<br>(21.93–23.38)	26.27<br>(25.5–27.05)	32.79<br>(31.92–33.66)	36.64<br>(35.72–37.56)
**Third definition**							
Overall		42.44<br>(41.87–43.01)	46.43<br>(45.83–47.03)	49.90<br>(49.28–50.52)	53.74<br>(53.1–54.39)	66.55<br>(65.83–67.27)	74.20<br>(73.44–74.96)
Sex	*Female*	69.12<br>(58.88–79.36)	75.52<br>(64.92–86.12)	81.39<br>(70.49–92.29)	86.68<br>(75.49–97.88)	105.88<br>(93.55–118.2)	115.68<br>(102.31–129.0)
	Male	14.57<br>(9.52–19.61)	16.02<br>(10.79–21.25)	16.61<br>(11.33–21.89)	17.80<br>(12.35–23.25)	22.57<br>(16.43–28.7)	26.58<br>(19.62–33.53)
Age	*18–29*	27.63<br>(14.09–41.16)	27.04<br>(13.79–40.28)	28.32<br>(14.86–41.78)	31.83<br>(17.52–46.14)	40.88<br>(24.53–57.24)	46.38<br>(27.83–64.93)
	30–39	56.54<br>(36.95–76.12)	60.44<br>(40.13–80.76)	64.71<br>(43.58–85.84)	71.50<br>(49.07–93.93)	82.68<br>(58.26–107.1)	83.21<br>(57.11–109.32)
	40–49	56.47<br>(40.66–72.28)	66.04<br>(48.9–83.18)	74.27<br>(55.94–92.6)	87.58<br>(67.36–107.8)	112.24<br>(88.94–135.5)	131.4<br>(104.42–158.4)
	50–59	54.88	60.32	59.79	64.10	79.67	86.07

Variable		2017	2018	2019	2020	2021	2022
		(39.67–70.08)	(44.67–75.98)	(44.41–75.17)	(48.27–79.92)	(62.11–97.23)	(67.1–105.05)
	60–69	41.06 (26.61–55.51)	47.94 (32.5–63.39)	52.73 (36.79–68.67)	53.98 (38.04–69.93)	66.61 (49.17–84.05)	82.03 (62.25–101.82)
	70–79	32.13 (18.05–46.21)	30.88 (17.35–44.41)	37.42 (22.76–52.09)	36.55 (22.22–50.87)	48.35 (32.1–64.59)	48.94 (32.25–65.64)
	80+	21.07 (7.31–34.84)	22.35 (8.5–36.21)	23.03 (9.42–36.63)	17.65 (6.12–29.18)	17.01 (5.9–28.12)	21.45 (8.77–34.12)
Geographical area	*Northern Italy*	50.23 (49.32–51.15)	53.1 (52.16–54.04)	56.71 (55.74–57.69)	61.04 (60.03–62.05)	75.94 (74.82–77.06)	84.96 (83.78–86.15)
	Central Italy	42.42 (41.14–43.71)	49.07 (47.69–50.45)	53.18 (51.74–54.61)	56.32 (54.85–57.8)	69.65 (68–71.29)	75.11 (73.41–76.82)
	Southern Italy & Islands	28.76 (27.95–29.57)	31.29 (30.44–32.14)	33.82 (32.94–34.7)	37.40 (36.48–38.33)	45.55 (44.53–46.57)	54.41 (53.29–55.53)

Figure S1. Graphical algorithm depicting the three different SLE definitions.

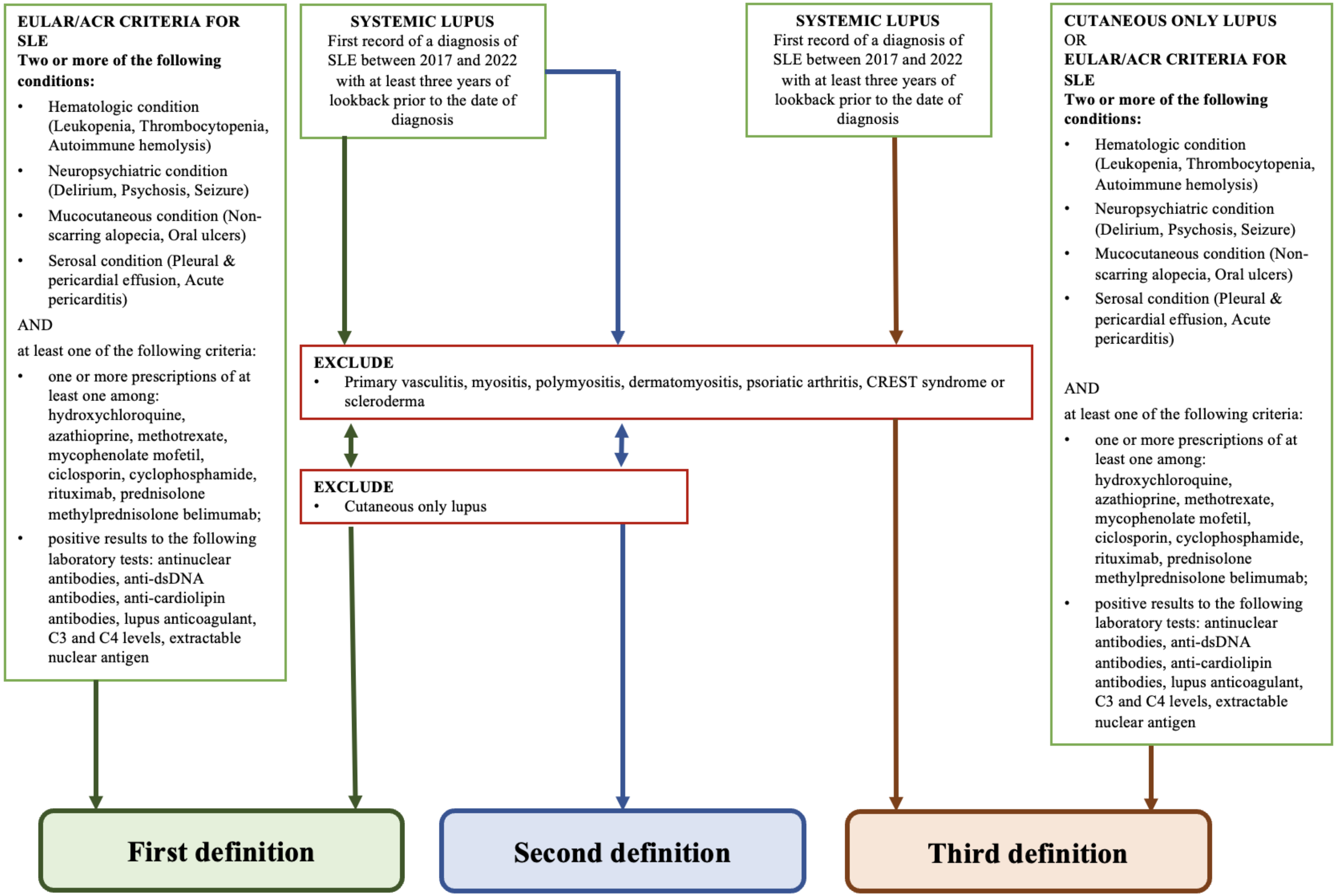

Supplement: online supplemental file 1 [file lupus-11-1-s001.pdf]
